# Serum autoantibody profiles in cancer patients treated with immune checkpoint inhibitors: a cross-sectional study

**DOI:** 10.3389/fonc.2025.1716014

**Published:** 2025-11-24

**Authors:** Kun Chen, Bao’e Guo, Rufeng Liu, Juan Wang, Chen Zhang, Guobing Xu

**Affiliations:** Key Laboratory of Carcinogenesis and Translational Research (Ministry of Education), Department of Clinical Laboratory, Peking University Cancer Hospital & Institute, Beijing, China

**Keywords:** autoantibody, antinuclear antibody, cancers, immune checkpoint inhibitor, immunotherapy

## Abstract

**Background:**

The relationship between autoantibody profiles and immune checkpoint inhibitor (ICI) therapy in cancer patients remains incompletely characterized. This cross-sectional study investigated serum autoantibody (AAb) prevalence and profiles across multiple tumor types before and after ICI therapy.

**Methods:**

This study analyzed serum autoantibodies in 808 participants: 358 treatment-naïve cancer patients (ICI- group), 250 cancer patients following ≥6 months of ICI therapy (ICI+ group), and 200 healthy controls (HC). The cancer cohort comprised 10 solid tumor types. Serum samples were analyzed for antinuclear antibodies (ANA), antiphospholipid antibodies (aPL), anti-neutrophil cytoplasmic antibodies (ANCA), and anti-thyroid antibodies (ATA) using automated quantitative immunoassays.

**Results:**

Cancer patients demonstrated significantly elevated AAb prevalence compared to healthy controls. The ICI− group showed positivity rates of 20.1% for ANA, 11.5% for aPL, 1.7% for ANCA, and 17.3% for ATA, compared to 10.5%, 4.5%, 1.5%, and 9.5% in healthy controls, respectively. After ICI therapy, ANA positivity increased to 33.6%. CTLA-4 inhibitor recipients demonstrated higher ANA frequencies than PD-1/PD-L1 monotherapy recipients (57.1% vs 27.1%),. Tumor stage did not significantly influence AAb prevalence. Colorectal, hepatocellular, and renal cancers showed significant ANA increases after ICI treatment. Anti-Scl-70, anti-SSA-60, and anti-RNP were the most frequently elevated ANA subtypes. Anti-thyroglobulin was the most responsive ATA subtype following ICI therapy.

**Conclusions:**

ANA profiles vary across tumor types and differ between treatment-naïve and ICI-treated patients. CTLA-4-treated patients exhibit higher ANA frequency. Different tumors exhibit distinct preferences for AAb expression patterns. Serum AAb profiling may serve as a valuable tool for immunotherapy monitoring and risk stratification for immune-related adverse events.

## 1 Introduction

Serum autoantibodies (AAb) are pivotal diagnostic markers for autoimmune disorders. Over recent decades, the complex relationship between autoimmunity and malignancy has gained attention, as cancer patients frequently develop humoral immune responses against self-antigens (1, 2). Studies demonstrate significantly elevated AAb levels, particularly antinuclear antibodies (ANA), in cancer patients, although the underlying mechanisms remain incompletely understood (3–5).

Immune checkpoint inhibitors (ICIs) have revolutionized cancer therapy and are now guideline-recommended first-line treatments for multiple malignancies (6, 7). ICIs are a class of anticancer drugs that work by releasing the “brakes” on the immune system through interaction with co-inhibitory and co-stimulatory molecules to regulate T cell activation. PD-1/PD-L1 blockade primarily affects peripheral tolerance, while CTLA-4 inhibition disrupts T-cell priming in lymph nodes (8–10). However, this immune activation can trigger immune-related adverse events (irAEs) through nonspecific immune system stimulation affecting virtually all organ systems. Previous studies suggested that AAb may serve as prognostic factors for non-small-cell lung cancer patients initiating immuno-therapy (11), with AAb occurrence associated with favorable prognosis and irAE prediction in certain cancers (12–15).

Despite these observations, the specific roles of different AAb subtypes in tumor development, progression, and immunotherapy response across various cancer types remain poorly defined. Available data on baseline AAb prevalence and immunotherapy-associated changes are incomplete or sometimes contradictory (12, 16, 17). While indirect immunofluorescence assay (IFA) using HEp-2 cells remains the American College of Rheumatology-recommended method for ANA screening, the interpretation of semi-quantitative AAb results is often complicated by interobserver variability and different cutoff values in clinical applications (18). Systematic research on baseline and post-immunotherapy AAb prevalence across multiple tumor types is lacking.

This cross-sectional study aimed to comprehensively evaluate AAb types and positivity rates in patients with different solid tumors by comparing treatment-naïve patients with those following immunotherapy using automated quantitative methods. Additionally, we explored potential associations between AAb profiles and clinical-pathological characteristics to provide essential data for efficacy monitoring and irAEs risk stratification in cancer immunotherapy.

## 2 Materials and methods

### 2.1 Study design and participants

This cross-sectional study examined AAb types and levels in healthy individuals, treatment-naïve cancer patients, and cancer patients following at least 6 months of immunotherapy. The study was conducted from March to April 2023 at Peking University Cancer Hospital. The control population comprised 200 healthy individuals (91 males, 109 females; median age 49 years, range 23-71) undergoing routine physical examinations without evidence of malignancy or autoimmune disease. The treatment-naïve cohort included 358 hospitalized cancer patients (160 males, 198 females; age ranges 18–88 and 16–82 years, respectively) without prior therapy. The post-immunotherapy cohort comprised 250 hospitalized cancer patients who had received ICI therapy for at least 6 months or completed a minimum of 6 treatment cycles, whichever occurred first. This dual criterion accommodates different dosing schedules. The cancer types included: gastric, colorectal, esophageal, hepatocellular, lung, breast, cervical, renal, lymphoma, and melanoma. Inclusion criteria were: (1) histologically confirmed cancer diagnosis; (2) for the ICI+ group, completion of at least 6 months or 6 cycles of ICI therapy. Exclusion criteria included: (1) pre-existing autoimmune diseases or conditions affecting AAb levels; (2) pregnancy, HIV positivity, systemic infection, or blood transfusion within 60 days. Due to the cross-sectional design, pre- and post-immunotherapy cohorts were independent groups sampled contemporaneously rather than matched pairs.

All patients’ sera were examined for antinuclear antibodies (ANA), antiphospholipid antibodies (aPL), anti-neutrophil cytoplasmic antibodies (ANCA), and anti-thyroid antibodies (ATA). Sera were collected from the Department of Clinical Laboratory (Peking University Cancer Hospital) with informed consent. Blood samples were collected in serum tubes from antecubital veins and frozen at −20 °C before use. Sera underwent no more than two freeze-thaw cycles.

Clinical and laboratory data were retrospectively collected from electronic medical records, including age, sex, underlying conditions, histological type, and TNM classification.

### 2.2 Serum autoantibodies analysis

Sixteen ANA subtypes were measured using a Luminex-based multiplex assay (Tesmi-F4000; Tellgen^®^, Shanghai, China): anti-SSB, SSA, Ro-52(SSA-52), Sm, Scl-70, RNP, Ribosomal P, PM/Scl, PCNA, Nucleosome, AMA-M2, Jo-1, Histone, dsDNA, Centromere B, and C1q. Antigens were covalently cross-linked to fluorescence-coded microspheres. Goat anti-human IgG antibody-labeled phycoerythrin was added, then microspheres were then resuspended in sheath fluid. The manufacturer’s recommended cut-off for positivity was >20 arbitrary units (AU) for all antibodies except anti-dsDNA (>20 IU/mL) and C1q (>10 U/mL).

IgG, IgM, and IgA antibodies against cardiolipin (CL) and β2-glycoprotein I were measured using fully automated chemiluminescence immunoassay (iFlash 3000, YHLO Biotech, Shenzhen, China). Anti-myeloperoxidase (MPO IgG), anti-proteinase 3 (PR3 IgG), and anti-glomerular basement membrane (GBM IgG) were measured using the same platform with manufacturer-recommended cutoffs.

Antithyroid antibodies (ATA), including Anti-TSH receptor (A-TSHR), anti-thyroglobulin (A-TG), and anti-thyroid peroxidase (A-TPO), were measured using electrochemiluminescence immunoassay (Elecsys and Cobas e 602 analyzers, Roche Diagnostics GmbH, Germany). Positivity thresholds were: A-TSHR >1.22 IU/L, A-TPO >34 IU/mL, and A-TG >115 IU/mL. All AAb were measured at the clinical laboratory of medicine in Peking University Cancer Hospital.

All autoantibody assays underwent rigorous quality control procedures. For the Luminex-based multiplex ANA panel, we analyzed quality control materials provided by the manufacturer at the beginning of each testing batch. For chemiluminescence assays (aPL and ANCA), we used commercial quality control materials with established target ranges. Inter-assay CV was <5% for all analytes. For anti-thyroid antibodies measured by electrochemiluminescence, quality control samples were tested daily, with acceptance criteria of ±8% of target values. All quality control results were within acceptable ranges throughout the study period.

### 2.3 Statistical analysis

Logistic regression analysis was performed to adjust for age, sex, and clinical tumor staging. Qualitative variables were expressed as numbers and percentages. Quantitative variables were presented as means (± standard deviation) for normal distributions or medians (interquartile ranges) for non-normal distributions. Group comparisons utilized Student’s t-test or Mann-Whitney U test as appropriate. Frequencies were compared using Fisher’s exact test or Chi-square test. To account for multiple comparisons, we applied Bonferroni correction in comparing four AAb categories (ANA, aPL, ANCA, ATA) between three groups (HC, ICI-, ICI+). Statistical analyses were performed using SPSS software (version 26.0, IBM Corp., Armonk, NY, USA), with figures created using GraphPad Prism (version 9.0, La Jolla, CA, USA). Two-tailed *P* values <0.05 were considered statistically significant.

## 3 Results

### 3.1 Patient characteristics

We enrolled 358 treatment-naïve cancer patients (ICI- patients) and 250 cancer patients who had received at least 6 months of ICI therapy (ICI+ patients). Additionally, we collected serum samples from 200 healthy individuals undergoing routine physical examinations during the same period as controls. The two cancer patient groups showed no significant differences in gender distribution or age (*P*>0.05). The cancer types and their respective frequencies in both patient groups are presented in **Table 1**.

**Table 1:** Clinical and laboratory characteristics of the 358 cancer patients (ICI-) and 250 cancer patients after immunotherapy (ICI+).

Characteristics	ICI- patients	ICI+ patients	*P* value
Males/Females	160/198	101/149	0.293
Age (yr)*	58.4 ± 12.9 (16-88)	60.2 ± 11.6 (18-89)	0.119
Cancer involving organs	Number of patients (%)		
Stomach	50 (14.0)	33 (13.2)	
Colorectal	50 (14.0)	31 (12.4)	
Esophageal	30 (8.4)	22 (8.8)	
Liver	30 (8.4)	19 (7.6)	
Lung	50 (14.0)	31 (12.4)	
Breast	23 (6.4)	14 (5.6)	
Cervic	19 (5.3)	18 (7.2)	
Kidney	28 (7.8)	13 (5.2)	
Lymphoma	38 (10.6)	32 (12.8)	
Melanoma	40 (11.2)	37 (14.8)	
Cancer stages			
Early stage (I/II)	104 (29.0)	18 (7.2)	<0.001
Advanced	233 (65.1)	220 (88)	
Unknown	21 (5.9)	12 (4.8)	

*mean ± s.d. (range).

### 3.2 Relationship between AAb prevalence, tumor stage, and ICI type

We investigated the AAb profiles in patients with tumors at different stages receiving ICIs. As shown in **Table 2**, although no statistical significance was observed, patients with advanced-stage tumors (stages III/IV) exhibited increased ANA positivity rates (34.1%) and slightly decreased ANCA positivity rates (2.7%) compared to those with early-stage tumors (stages I/II), while aPL and ATA showed no notable changes. These findings suggest that tumor stage may not critically influence AAb formation.

**Table 2:** The prevalence of autoantibodies in immunotherapy patients across different cancer stages n (%).

AAb types	I/II (*n* = 18)	III/IV (*n* = 220)	*P* value
ANA	4 (22.2)	75 (34.1)	0.304
aPL	2 (11.1)	22 (10.0)	1.0
ANCA	1 (5.6)	6 (2.7)	0.428
ATA	4 (22.2)	47 (21.4)	1.0

We examined AAb profiles according to ICI types (**Table 3**). ANA positivity was significantly higher in patients receiving CTLA-4 monotherapy (57.1%) or combination therapy (51.4%) compared to PD-1/PD-L1 monotherapy (27.6%; *P* = 0.005 and *P* = 0.002, respectively). No significant differences were observed in aPL, ANCA, or ATA positivity across treatment groups.

**Table 3:** The prevalence of autoantibodies in cancer patients treated with different immunotherapy drugs n (%).

AAb types	CTLA-4 (*n* = 21)		PD-1/PD-L1+CTLA4 (*n* = 37)		PD-1/PD-L1 (*n* = 192)
	Pos.	*P* value	Pos.	*P* value	Pos.
ANA	12 (57.1)	0.005	20 (54.1)	0.002	52 (27.1)
aPL	2 (9.5)	1.0	3 (8.1)	1.0	21 (10.9)
ANCA	0 (0)	1.0	1 (2.7)	1.0	5 (2.6)
ATA	5 (23.8)	0.795	8 (21.6)	0.971	40 (20.8)

*P* < 0.05, CTLA-4 *vs* PD-1/PD-L1, and PD-1/PD-L1+CTLA4 *vs* PD-1/PD-L1, respectively.

### 3.3 AAb Prevalence in ICI−, ICI+, and control groups

In healthy controls (HC), baseline positivity rates were 10.5% for ANA, 4.5% for aPL, 1.5% for ANCA, and 9.5% for ATA, which are comparable to published data and confirm that our detection system accurately assessed serum AAb (**Table 4**) (19–22). Treatment-naïve cancer patients showed significantly elevated rates: 20.1% for ANA (*P* = 0.003), 11.5% for aPL (*P* = 0.006), 1.7% for ANCA (*P* = 1.0), and 17.3% for ATA (*P* = 0.012), suggesting tumor-associated B-cell activation or autoimmune dysregulation. In ICI+ group, ANA positivity increased to 33.6% (*P* < 0.001 vs ICI- group), while aPL, ANCA, and ATA showed no significant changes. Among ANA subtypes, the five most prevalent in cancer patients were anti-SSA-60, anti-Scl-70, anti-RNP, anti-Nucleosome, and anti-AMA-M2. Anti-Scl-70 showed significant post-immunotherapy elevation (8.8% vs 4.5%, *P* = 0.03).

**Table 4:** Prevalence of the autoantibodies in cancer patients without any treatment (ICI-), cancer patients after at least 6-month immunotherapy (ICI+), and healthy controls (HC) n (%).

AAb types	ICI- (*n* = 358)		ICI+ (*n* = 250)		HC (*n* = 200)
	Pos.	*P* value	Pos.	*P* value	Pos.
ANAs	**72 (20.1)**	**0.003**	**84 (33.6)**	**<0.001**	**21 (10.5)**
SSB	2 (0.6)	0.539	4 (1.6)	0.389	0 (0)
SSA-60	14 (3.9)	0.048	18 (7.2)	0.074	2 (1.0)
Ro-52	7 (2.0)	0.955	4 (1.6)	0.989	3 (1.5)
Sm	0 (0)	0.358	1 (0.4)	0.411	1 (0.5)
Scl-70	16 (4.5)	0.132	22 (8.8)	0.03	4 (2)
RNP	18 (5.0)	0.036	16 (6.4)	0.469	3 (1.5)
Ribosomal P	3 (0.8)	0.487	3 (1.2)	0.978	0 (0)
PM/Scl	3 (0.8)	0.487	7 (2.8)	0.122	0 (0)
PCNA	0 (0)	—	0 (0)	—	0 (0)
Nucleosome	12 (3.4)	0.359	13 (5.2)	0.259	4 (2.0)
AMA-M2	11 (3.1)	0.453	8 (3.2)	0.929	4 (2.0)
Jo-1	1 (0.3)	1.0	2 (0.8)	0.754	0 (0)
Histone	0 (0)	0.011	2 (0.8)	0.169	5 (2.5)
dsDNA Centromere B	4 (1.1) 2 (0.6)	1.0 0.539	2 (0.8) 1 (0.4)	1.0 1.0	2 (1.0) 0 (0)
C1q	5 (1.4)	0.096	3 (1.2)	1.0	8 (4.0)
aPL	**41 (11.5)**	**0.006**	**26 (10.4)**	**0.683**	**9 (4.5)**
CL IgG	3 (0.8)	1.0	2 (0.8)	1.0	2 (1.0)
CL IgM	24 (6.7)	0.032	15 (6.0)	0.727	5 (2.5)
CL IgA	1 (0.3)	1.0	0 (0)	1.0	0 (0)
β2-GPI IgG	0 (0)	—	1 (0.4)	0.411	0 (0)
β2-GPI IgM	38 (10.6)	0.003	18 (7.2)	0.152	7 (3.5)
β2-GPI IgA	0 (0)	—	0 (0)	—	0 (0)
ANCA	**6 (1.7)**	**1.0**	**6 (2.4)**	**0.737**	**3 (1.5)**
MPO IgG	4 (1.1)	1.0	3 (1.2)	1.0	2 (1.0)
PR3 IgG	1 (0.3)	1.0	2 (0.8)	0.754	1 (0.5)
GBM IgG	1 (0.3)	1.0	2 (0.8)	0.754	0 (0)
ATA	**62 (17.3)**	**0.012**	**53 (21.2)**	**0.229**	**19 (9.5)**
A-TG	32 (8.9)	0.425	36 (14.4)	0.036	14 (7.0)
A-TPO	28 (7.8)	0.131	20 (8.0)	0.936	9 (4.5)
A-TSHR	16 (4.5)	0.026	14 (5.6)	0.526	2 (1.0)

*P* < 0.05, ICI-*vs* HC, and ICI+ *vs* ICI-, respectively.

— too few case numbers to calculate.

Bold values indicated the total positive rates and P values of the four types of AAb.

Within the aPL profile, CL IgM (6.7%) and β2-GPI IgM (10.6%) showed the highest positivity rates in ICI- patients, significantly exceeding healthy controls (*P* = 0.032 and *P* = 0.003, respectively). Only one patient tested positive for CL IgA, while all β2-GPI IgA tests were negative. No significant differences in aPL profiles were observed between ICI- and ICI+ groups.

ANCA positivity in ICI− patients was low (1.7%), showing no statistical difference from controls or ICI+ patients. ATA positivity reached 17.3% in ICI− patients, higher than controls (9.5%, *P* = 0.012). Total ATA positivity showed an upward trend after immunotherapy (21.2%), with only A-TG reaching statistical significance (14.4%, *P* = 0.036), while A-TPO and A-TSHR remained unchanged.

### 3.4 Prevalence of AAb in patients with different types of cancers

**Figure 1:**
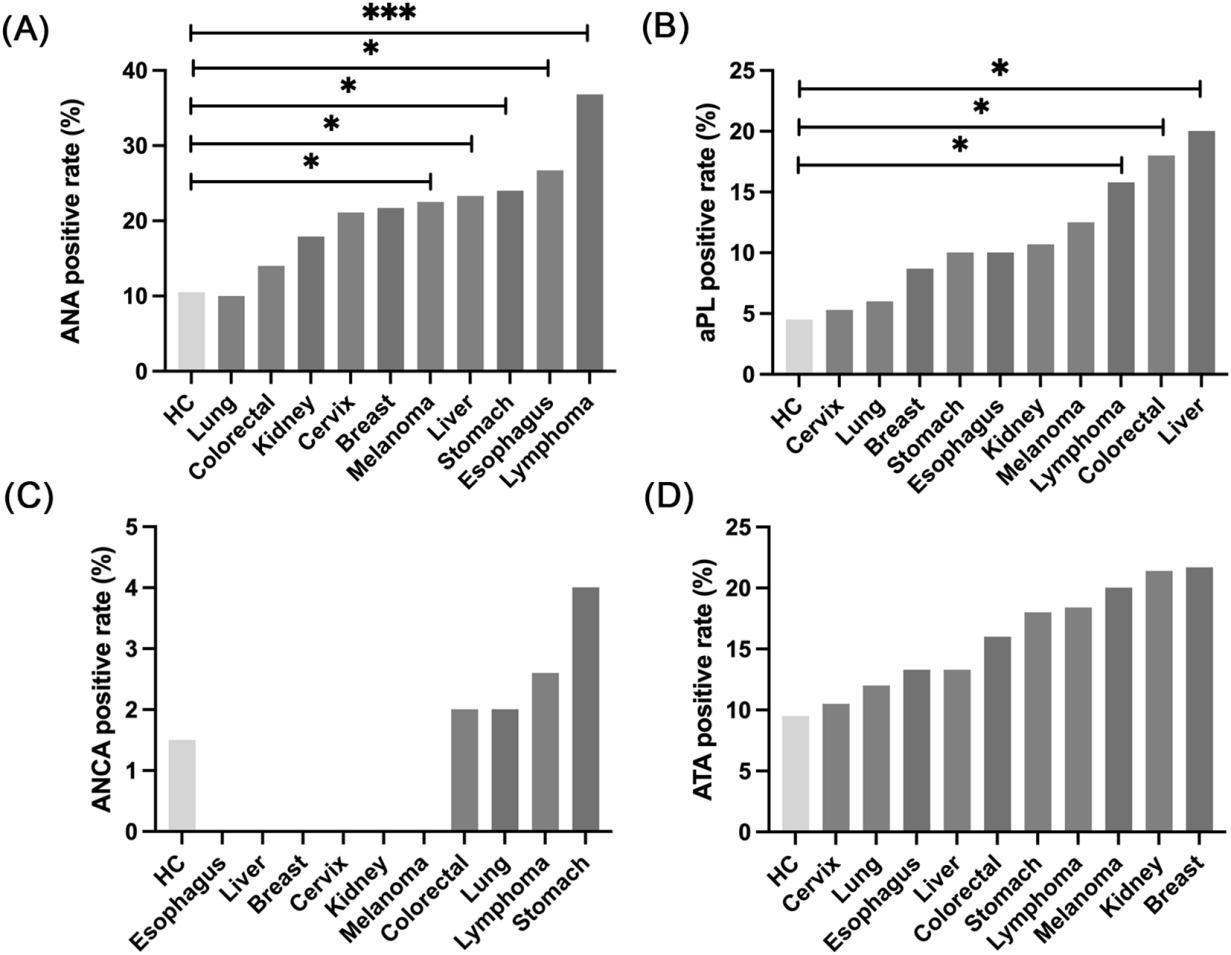
Prevalence of autoantibodies among treatment-naïve cancer patients. **(A)** Comparison of ANA positivity rates between patients with different cancer types and healthy controls. Significant higher positivity rates were observed in patients with melanoma, hepatocellular carcinoma, gastric cancer, esophageal cancer, and lymphoma compared to healthy controls. **(B)** Comparison of aPL positivity rates between patients with different cancer types and healthy controls. Patients with lymphoma, colorectal cancer, and hepatocellular carcinoma exhibited significantly higher positivity rates compared to healthy controls. **(C)** Comparison of ANCA positivity rates between patients with different cancer types and healthy controls. Patients with colorectal cancer, lung cancer, lymphoma, and gastric cancer demonstrated significantly higher positivity rates compared to healthy controls. ANCA positivity was not detected in patients with esophageal cancer, hepatocellular carcinoma, breast cancer, cervical cancer, renal cell carcinoma, or melanoma. **(D)** Comparison of ATA positivity rates between patients with different cancer types and healthy controls. All cancer types exhibited significantly higher ATA positivity rates compared to healthy controls. *P < 0.05; ***P < 0.001.

We investigated whether different tumor types exhibit distinct antibody distribution patterns by comparing AAb prevalence between ICI− patients and healthy volunteers. Nearly all tumor types showed increased AAb generation. Patients with melanoma, hepatocellular carcinoma, gastric cancer, esophageal cancer, and lymphoma demonstrated significantly higher ANA positivity (*P* < 0.05). Notably, lymphoma exhibited an ANA positivity rate of 36.8%, substantially higher than other tumors, suggesting increased lymphocyte antigen epitope activation or autoimmune phenomena (**Figure 1A**). For aPL, lymphoma (15.8%), colorectal cancer (18%), and hepatocellular carcinoma (20%) showed significantly elevated rates compared to healthy controls (**Figure 1B**). ANCA positivity was generally low, with marginal elevations only in colorectal cancer, lung cancer, lymphoma, and gastric cancer (**Figure 1C**). ATA positivity in ICI− patients was elevated compared to controls, though not significantly (**Figure 1D**).

Comparison between ICI- and ICI+ groups revealed significant ANA increases in colorectal cancer (32.3%, *P* = 0.049), hepatocellular carcinoma (52.6%, *P* = 0.036), and renal cell carcinoma (53.8%, *P* = 0.047) compared with the ICI- group (**Table 5**). All tumor types except gastric cancer showed increased ANA positivity following immunotherapy. aPL and ANCA profiles remained largely unchanged, while ATA positivity increased in 9 of 10 tumor types (excluding melanoma), though without statistical significance.

**Table 5:** The impact of immunotherapy on autoantibody positivity rates across different tumor types n (%).

Cancer	ANAs			aPL			ANCA			ATA		
	ICI-	ICI+	*P*	ICI-	ICI+	*P*	ICI-	ICI+	*P*	ICI-	ICI+	*P*
Stomach	12 (24)	7 (21.2)	0.767	5 (10)	4 (12.1)	1.0	2 (4)	2 (6.1)	1.0	9 (18)	7 (21.2)	0.717
Colorectal	7 (14)	10 (32.3)	0.049	9 (18)	2 (6.5)	0.254	1 (2)	0 (0)	1.0	8 (16)	7 (22.6)	0.459
Esophagus	8 (26.7)	6 (27.3)	0.961	3 (10)	3 (13.6)	1.0	0 (0)	1 (4.5)	0.423	4 (13.3)	5 (22.7)	0.607
liver	7 (23.3)	10 (52.6)	0.036	6 (20)	4 (21.1)	1.0	0 (0)	1 (5.3)	0.388	4 (13.3)	5 (26.3)	0.444
Lung	5 (10)	8 (25.8)	0.116	3 (6)	3 (9.7)	0.859	1 (2)	1 (3.2)	1.0	6 (12)	6 (19.4)	0.559
Breast	5 (21.7)	5 (35.7)	0.454	2 (8.7)	1 (7.1)	1.0	0 (0)	0 (0)	—	5 (21.7)	5 (35.7)	0.454
Cervix	4 (21.1)	5 (27.8)	0.714	1 (5.3)	0 (0)	1.0	0 (0)	0 (0)	—	2 (10.5)	3 (16.7)	0.660
Kidney	5 (17.9)	7 (53.8)	0.047	3 (10.7)	1 (7.7)	1.0	0 (0)	0 (0)	—	6 (21.4)	3 (23.1)	1.0
Lymphoma	14 (36.8)	13 (40.6)	0.746	6 (15.8)	8 (25.0)	0.337	1 (2.6)	0 (0)	1.0	7 (18.4)	6 (18.8)	0.972
Melanoma	9 (22.5)	12 (32.4)	0.328	5 (12.5)	3 (8.1)	0.797	0 (0)	1 (2.7)	0.481	8 (20.0)	6 (16.2)	0.667

— too few case numbers to calculate.

**Figure 2:**
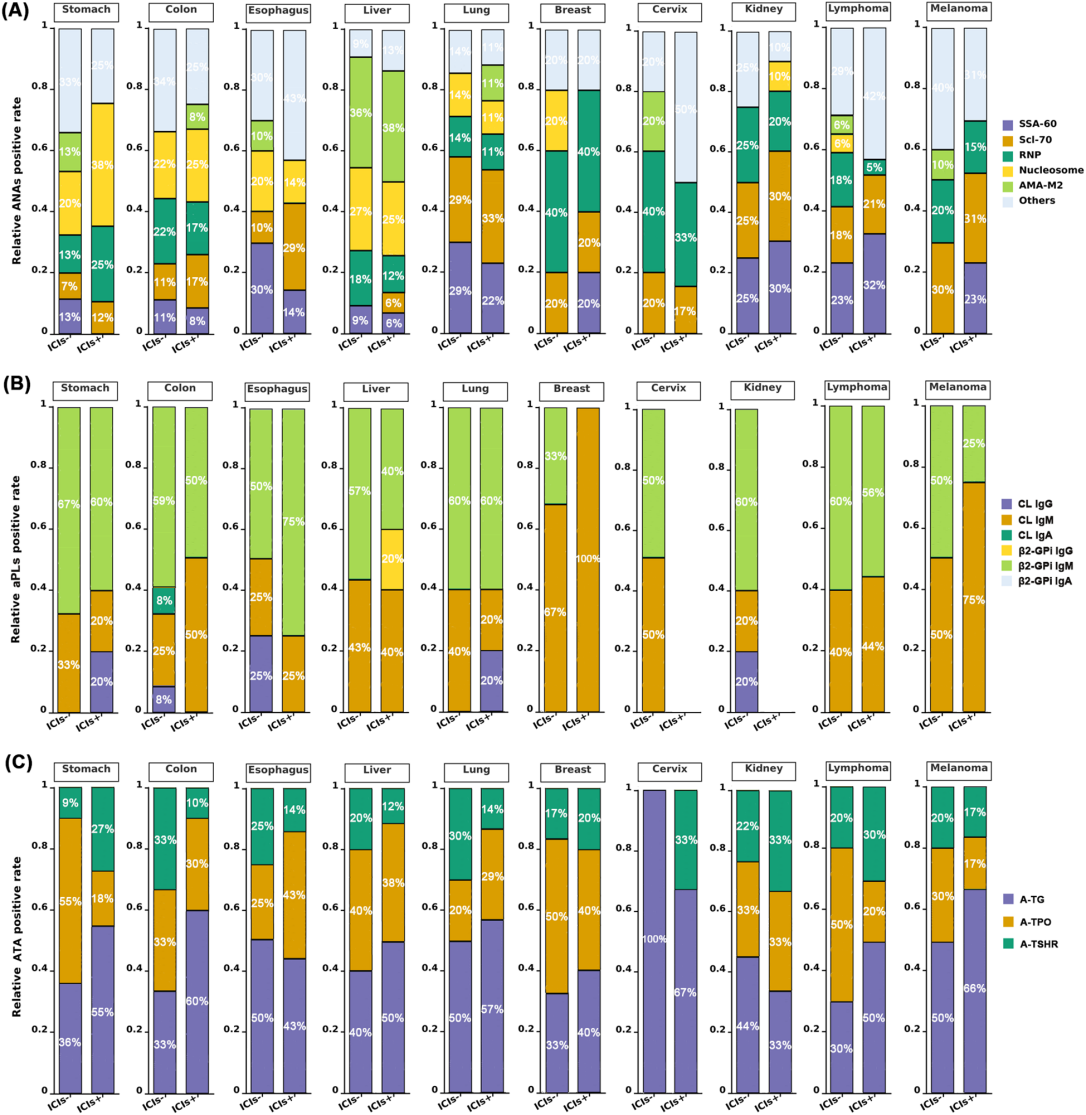
Effects of immunotherapy on autoantibody subtype positivity patterns in cancer patients. **(A)** The five most frequently detected autoantibodies from the ANA panel in cancer patients were selected to analyze ANA positivity patterns across different tumor types before and after immunotherapy. Remaining ANA specificities were categorized as “others”. **(B)** Analysis of aPL positivity patterns across different tumor types before and after immunotherapy demonstrated that IgM was the predominant isotype for both anti-cardiolipin and anti-β2-glycoprotein I antibodies. **(C)** Analysis of ATA positivity patterns across different tumor types before and after immunotherapy showed that anti-TG and anti-TPO exhibited higher positivity rates, with increased anti-TG positivity observed following immunotherapy in most tumor types.

Interestingly, analysis of the five most prevalent ANA subtypes across 10 tumors revealed tumor-specific expression patterns. Anti-SSA-60 showed the highest positivity in renal cell carcinoma, lymphoma, and lung cancer. Anti-Scl-70, while broadly expressed, predominated in lung cancer, renal cell carcinoma, and melanoma (approximately 30% of ANA-positive cases). Anti-RNP showed highest rates in breast and cervical cancers (approximately 33-40%). Anti-Nucleosome preferentially occurred in gastrointestinal tumors, esophageal cancer, and hepatocellular carcinoma. High anti-AMA-M2 positivity (approximately 36-38%) in hepatocellular carcinoma suggests hepatic tissue destruction may induce hepatic autoimmunity (**Figure 2A**).

AAb for Cardiolipin and β2-Glycoprotein I in various tumor types were predominantly IgM subtype (approximately 20%-100%), followed by IgG (8-25%). IgA was least frequent, with only one CL IgA-positive case in an untreated colorectal cancer patient. aPL distribution varied considerably among tumor types, with no positive cases in immunotherapy-treated cervical and renal cancer patients (**Figure 2B**).

Among thyroid-associated AAb, A-TG demonstrated the most significant treatment-related changes. A-TG positivity was significantly elevated in ICI+ patients compared to untreated controls across all tumor types. Although this difference was less pronounced when stratified by individual tumor types, A-TG maintained the highest positive expression rate across most malignancies. Following immunotherapy, A-TG positivity increased in all tumor types except esophageal, cervical, and renal cancers. Conversely, A-TPO prevalence decreased in multiple tumor types receiving immuno-therapy, including gastrointestinal tumors, hepatocellular carcinoma, breast cancer, lymphoma, and melanoma. This inverse relationship between A-TG and A-TPO suggests that A-TG may represent the most clinically relevant thyroid AAb requiring post-immunotherapy monitoring. A-TSHR positivity was significantly higher in cancer patients compared to healthy controls (*P* = 0.026). However, among immunotherapy-treated patients, A-TSHR did not exhibit consistent patterns, with approximately half of the tumor types showing increased expression (**Figure 2C**). ANCA showed minimal positivity across cancer patients and was excluded from detailed analysis due to limited sample size and potential analytical bias.

## 4 Discussion

Elevated AAb prevalence in cancer patients has been well-documented over recent decades (23–25), reflecting profound immune system remodeling and potentially predicting treatment response and irAEs (13, 26, 27). However, the dynamic serum AAb patterns during ICI therapy remain complex and controversial, possibly due to differential clinical significance across cancer types and methodological variations. We employed automated Luminex multiplex and chemiluminescent immunoassays to systematically evaluate AAb profiles before and after immunotherapy across multiple solid tumors. To our knowledge, this is the first study to investigate AAb prevalence changes following immunotherapy across multiple cancer types.

The mechanisms underlying elevated baseline AAb positivity in cancer patients are multifactorial. Tumor-associated antigens (TAAs) constitute the primary drivers, as cancer cells express aberrant proteins through oncogene activation, tumor suppressor inactivation, and abnormal post-translational modifications (28). These altered proteins, including overexpressed nucleophosmin, nucleolin, and mutant p53, become immunogenic targets that cross-react with normal cellular components. Additionally, the tumor microenvironment promotes autoimmunity through chronic inflammation and extensive cell death. Apoptotic and necrotic tumor cells release sequestered nuclear antigens, breaking immunological tolerance and exposing cryptic self-antigens (29). Inflammatory cytokines (TNF-α, IL-1, IL-6) at tumor sites create a pro-autoimmune milieu where the immune system generates AAb against tumor-associated targets, potentially increasing autoimmune risk (30). Dual BCR/TLR7 signaling promotes autoantibody-producing plasma cell differentiation, which correlates with antitumor responses and may explain the frequent association between immunotherapy efficacy and autoimmune toxicity (13, 31).

Our finding that AAb frequency remained independent of tumor staging suggests that tumor burden may not critically influence AAb formation, consistent with previous lymphoma studies (32). However, the type of ICI significantly influenced ANA formation. Patients receiving CTLA-4 inhibitors, alone or combined with PD-1/PD-L1 inhibitors, demonstrated significantly higher ANA frequencies compared to PD-1/PD-L1 mono-therapy. This differential effect reflects distinct immunological mechanisms. CTLA-4 blockade disrupts early T-cell activation checkpoints, causing broad immune activation and regulatory T-cell depletion, which removes peripheral tolerance mechanisms. This leads to enhanced T-B cell interactions, increased germinal center formation, and direct B-cell activation (31, 33, 34). Moreover, ipilimumab’s IgG1 structure enables complement activation and antibody-dependent cellular cytotoxicity, potentially causing tissue damage that releases autoantigens (35). Conversely, PD-1/PD-L1 inhibition primarily reverses late-stage T-cell exhaustion in peripheral tissues, producing more localized autoimmune reactions. The IgG4 structure of these antibodies results in reduced complement activation (36, 37).

The significantly elevated ANA prevalence in cancer patients likely represents a secondary phenomenon resulting from exposure to aberrant tumor antigens, rather than playing a direct role in tumorigenesis, though these antibodies may serve as valuable biomarkers (25). We identified five ANA subtypes with frequencies exceeding 3%: anti-AMA-M2, anti-Nucleosome, anti-SSA-60, anti-Scl-70, and anti-RNP, with anti-SSA-60 and anti-RNP significantly elevated compared to healthy controls. Anti-SSA-60 targets the cytoplasmic Ro60 protein involved in RNA metabolism, which undergoes dysregulation during tumorigenesis, resulting in enhanced immunogenicity (38). Anti-RNP antibodies target small nuclear ribonucleoproteins critical for pre-mRNA splicing, potentially released through tumor cell necrosis and apoptosis (39). Anti-Nucleosome antibody elevation correlates with massive nucleosome release during tumor cell apoptosis and necrosis processes (3, 40).

ICI+ patients demonstrated higher ANA detection frequency (33.6%) compared to ICI- patients (20.1%), with anti-Scl-70 showing significant elevation. Tumor-specific patterns emerged: melanoma, hepatocellular carcinoma, gastric cancer, esophageal cancer, and lymphoma demonstrated high baseline ANA frequencies, while colorectal, hepatocellular, and renal cancers showed significant post-immunotherapy increases. Distinct antibody expression patterns characterized different cancers; anti-AMA-M2 showed elevated frequencies in hepatocellular carcinoma both before and after immunotherapy, while anti-Nucleosome pre-dominated in gastrointestinal tumors. These findings suggest tumor-specific AAb panels may serve as potential biomarkers for monitoring therapeutic efficacy or predicting irAEs. Sample size limitations should be considered when interpreting tumor-specific findings. Several cancer types had relatively small sample sizes in the ICI+ group, including renal cell carcinoma (n=13) and breast cancer (n=14). While we observed statistically significant ANA increases in colorectal, hepatocellular, and renal cancers following ICI therapy, the limited sample sizes increase the risk of Type I errors and reduce statistical power to detect true differences. The observed effects may not be generalizable and require validation in larger cohorts.

aPL are primarily associated with thromboembolic events, a major cause of cancer-related mortality (22). Our data revealed that the prevalence of aPL in cancer patients was consistently elevated compared to healthy individuals. Specifically, patients with lymphoma, colorectal cancer, and hepatocellular carcinoma demonstrated significantly higher frequencies of aPL than healthy controls, with IgM aCL and IgM anti-β2-GPI showing highest prevalence. aPL development in cancer patients results from two synergistic mechanisms. First, tumor-mediated chronic inflammation produces AAb against phospholipid-binding proteins, particularly β2-glycoprotein I. Second, cancer cells present novel antigens that trigger cross-reactive antibody responses, with certain malignancies directly secreting aCL (41). Clonal B-cells lacking normal regulatory mechanisms undergo expansion, producing monoclonal immunoglobulins with aPL activity (42). Alcoholic liver disease and hepatitis B/C virus infections associated with liver cancer have also been shown to induce aPL formation (43–45). The coexistence of tumors and antiphospholipid syndrome (APS) should also be considered, as multiple studies have reported the association between advanced tumors and APS (46). Notably, aPL levels showed minimal variation between pre- and post-immunotherapy timepoints across tumor types, suggesting anti-tumor immune response reactivation may not significantly influence aPL expression. The IgA aCL and IgA β2-GPI occurred at relatively low frequencies in our cancer patient group, limiting our ability to assess their potential roles in tumorigenesis and immunotherapeutic responses.

ANCA prevalence in healthy individuals is relatively low, ranging from approximately 0% to 5.1% (47). Currently, large-scale studies directly comparing ANCA prevalence between cancer patients and healthy populations remain scarce. In this study, we observed slightly elevated ANCA frequency in cancer patients both before and after immunotherapy. Due to limited positive cases, the clinical significance of ANCA and its potential role in guiding immunotherapy decisions remain to be elucidated through larger-scale investigations.

ATA showed significantly elevated prevalence across nearly all tumor types compared to healthy controls, consistent with previous reports (27, 48, 49). Post-immunotherapy ATA frequency increased overall, with only A-TG reaching statistical significance. This reflects non-specific immune activation by immunotherapy, inducing autoimmune responses against vulnerable endocrine organs (50). The differential response between A-TG and A-TPO contrasts with some reports suggesting A-TPO as a potential irAEs biomarker, possibly due to variations in tumor types and therapeutic agents studied (51, 52).

Our findings raise important clinical questions regarding the role of AAb screening in ICI therapy management. Current evidence does not support routine pre-treatment AAb screening or treatment modification based solely on AAb elevations. Several studies demonstrate that patients with pre-existing autoimmune conditions or positive AAb can safely receive ICIs with appropriate monitoring (5, 12). However, AAb profiling may serve two potential clinical roles: risk stratification for immune-related adverse events (irAEs), with some evidence suggesting AAb-positive patients may have higher irAE risk (13–15, 27). Biomarker development for treatment response, as AAb development during therapy may correlate with anti-tumor efficacy (11, 12). Prospective studies are needed to establish whether AAb monitoring can guide personalized immunotherapy approaches, such as heightened irAE surveillance in high-risk patients or treatment optimization in AAb-positive responders. Until such evidence emerges, we recommend that ICI treatment decisions prioritize established clinical factors rather than AAb status alone, while remaining vigilant for clinical manifestations of autoimmunity.

Several limitations of our cross-sectional design warrant consideration. First, the observed differences in AAb prevalence between ICI- and ICI+ groups represent associations rather than causal relationships. Our study cannot definitively establish that ICIs directly cause AAb elevation, as alternative explanations exist: (1) patients who develop AAb may have differential treatment outcomes affecting their representation in the post-ICI cohort; (2) disease progression itself may influence AAb profiles independent of treatment; (3) time-dependent changes in immune status may confound comparisons between independently sampled cohorts. Prospective longitudinal studies following individual patients from treatment initiation through therapy would provide stronger evidence for causal relationships between ICI exposure and AAb changes. Second, we cannot exclude potential confounding effects from concurrent radiotherapy, chemotherapy, or other treatment modalities. Future investigations should establish multi-parameter risk assessment models based on continuous AAb profile dynamics. This approach could enhance prediction accuracy and facilitate high-risk patient identification and immunotherapy optimization.

In conclusion, our findings demonstrate that cancer patients exhibit increased ANA, aPL, and ATA frequencies compared to healthy individuals, with tumor-specific AAb preferences that are independent of disease staging. Immunotherapy further enhanced ANA frequency, particularly following CTLA-4 inhibitor administration. Among the four AAb profiles examined, ANA and A-TG demonstrated the most pronounced post-immunotherapy changes, while ANCA monitoring appears to have limited predictive value. Furthermore, different tumor types exhibit distinct ANA expression patterns. These distinct profiles across cancers suggest AAb may serve as potential biomarkers for predicting immunotherapy efficacy or irAEs development.

## Data Availability

The raw data supporting the conclusions of this article will be made available by the authors, without undue reservation.
